# Supplementary material for: Seminoma and Embryonal Carcinoma Footprints Identified by Analysis of Integrated Genome-Wide Epigenetic and Expression Profiles of Germ Cell Cancer Cell Lines
Source: PLoS One. 2014 Jun 2;9(6):e98330. doi: 10.1371/journal.pone.0098330 (PMC4041891; doi:10.1371/journal.pone.0098330)
Supplement: File S1 — ZIP file containing DMRforPairs output for significant regions. Please start from the html files. (ZIP) [file pone.0098330.s008.zip › figures/35304.pdf]

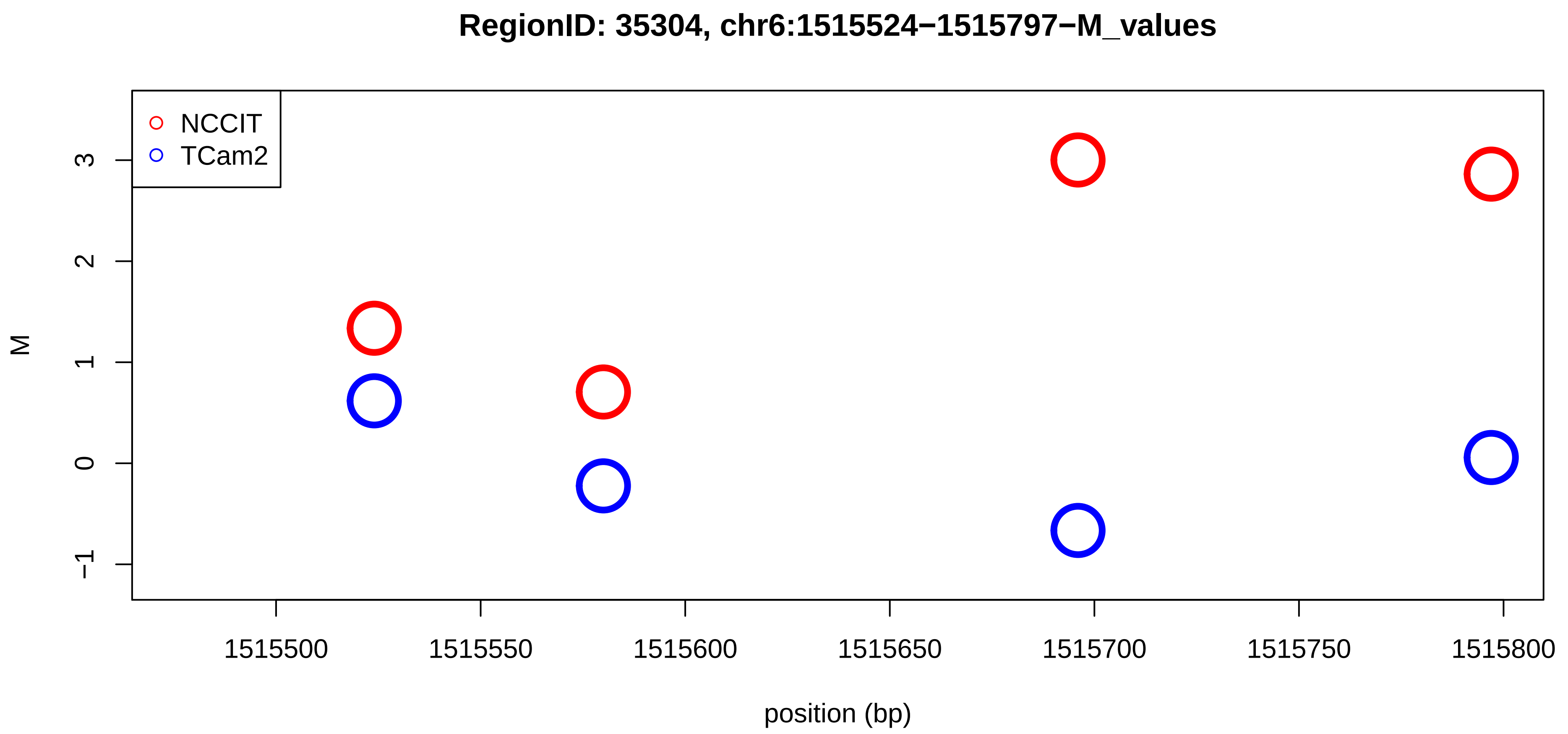
RegionID: 35304, chr6:1515524−1515797−M_values
NCCIT
TCam2
M
−1
0
1
2
3
1515500
1515550
1515600
1515650
1515700
1515750
1515800
position (bp)

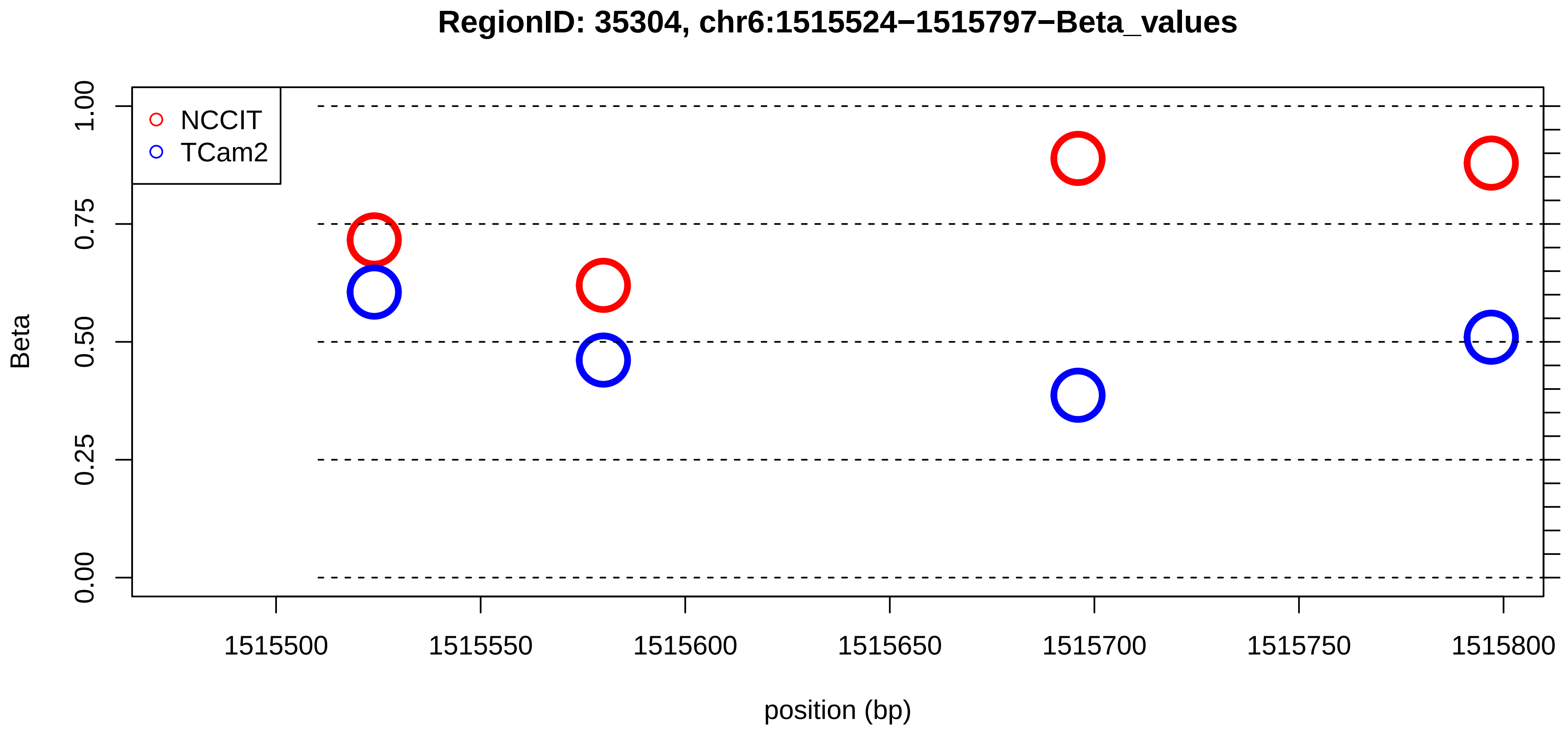
RegionID: 35304, chr6:1515524−1515797−Beta_values
NCCIT
TCam2
Beta
0.00
0.25
0.50
0.75
1.00
1515500
1515550
1515600
1515650
1515700
1515750
1515800
position (bp)
